# In Vitro and In Vivo Effects of Fermented Oyster-Derived Lactate on Exercise Endurance Indicators in Mice

**DOI:** 10.3390/ijerph17238811

**Published:** 2020-11-27

**Authors:** Storm N. S. Reid, Joung-Hyun Park, Yunsook Kim, Yi Sub Kwak, Byeong Hwan Jeon

**Affiliations:** 1 Department of Sports and Health Science, Kyungsung University, Busan 48434, Korea; stormreid29@gmail.com; 2 Marine Bio-Industry Development Center, Marine Bioprocesses Co., Ltd., Busan 46048, Korea; pdc327@hanmail.net (J.-H.P.); block0830@hanmail.net (Y.K.); 3 DEU-Exe-Physio Lab, Department of Physical Education, Dong-Eui University, Busan 47340, Korea; ysk2003@deu.ac.kr

**Keywords:** fermented oyster, functional foods, lactate, exercise endurance, mitochondrial biogenesis

## Abstract

Exogenous lactate administration has more recently been investigated for its various prophylactic effects. Lactate derived from potential functional foods, such as fermented oyster extract (FO), may emerge as a practical and effective method of consuming exogenous lactate. The current study endeavored to ascertain whether the lactate derived from FO may act on muscle cell biology, and to what extent this may translate into physical fitness improvements. We examined the effects of FO in vitro and in vivo, on mouse C2C12 cells and exercise performance indicators in mice, respectively. In vitro, biochemical analysis was carried out to determine the effects of FO on lactate content and muscle cell energy metabolism, including adenosine triphosphate (ATP) activity. Western blot analysis was also utilized to measure the protein expression of total adenosine monophosphate-activated protein kinase (AMPK), p-AMPK (Thr172), lactate dehydrogenase (LDH), succinate dehydrogenase (SDHA) and peroxisome proliferator-activated receptor gamma coactivator-1α (PGC-1α) in response to FO administration. Three experimental groups were formed: a positive control (PC) treated with 1% horse serum, FO10 treated with 10 μg/mL and FO50 treated with 50 μg/mL. In vivo, the effects of FO supplementation on exercise endurance were measured using the Rota-rod test, and Western blot analysis measured myosin heavy-chain 2 (MYH2) to assess skeletal muscle growth, alongside p-AMPK, total-AMPK, PGC-1α, cytochrome C and UCP3 protein expression. Biochemical analysis was also performed on muscle tissue to measure the changes in concentration of liver lactate, lactate dehydrogenase (LDH), glycogen and citrate. Five groups (n = 10/per group) consisted of a control group (CON), exercise group (Ex), positive control treated with Ex and 500 mg/kg Taurine (Ex-Tau), Ex and 100 mg/kg FO supplementation (Ex-FO100) and Ex and 200 mg/kg FO supplementation (Ex-FO200) orally administered over the 4-week experimental period.FO50 significantly increased PGC-1α expression (*p* < 0.001), whereas both FO10 and FO50 increased the expression of p-AMPK (*p* < 0.001), in C2C12 muscle cells, showing increased signaling important for mitochondrial metabolism and biogenesis. Muscle lactate levels were also significantly increased following FO10 (*p* < 0.05) and FO50 (*p* < 0.001). In vivo, muscle protein expression of p-AMPK (*p* < 0.05) and PGC-1α were increased, corroborating our in vitro results. Cytochrome C also significantly increased following FO200 intake. These results suggest that the effects of FO supplementation may manifest in a dose-response manner. FO administration, in vitro, and supplementation, in vivo, both demonstrate a potential for improvements in mitochondrial metabolism and biogenesis, and even for potentiating the adaptive effects of endurance exercise. Mechanistically, lactate may be an important molecule in explaining the aforementioned positive effects of FO.

## 1. Introduction

Regular physical activity is an established approach to improved quality of life and a preventative measure in the case of chronic and/or degenerative diseases. Exercise approaches, such as endurance training, have been shown to induce a number of advantageous physiological adaptations on skeletal muscle (SkM), including remodeling of the mitochondrial network, the contractile apparatus and the vasculatures [1,2], which underlie many of the health benefits. There are numerous factors that contribute to the aforementioned physiological adaptations, and lactate, previously considered a waste product of anaerobic metabolism, is currently identified as a valuable metabolic product. The more recent lactate paradigm suggests that it counteracts acidosis, maintains neuron and astrocyte function [3] and fuels aerobic metabolism. It is also an important intermediary in numerous metabolic processes [4].

One particular adaptation involves fluctuations in lactate concentrations, during prolonged exercise, resulting in the stimulation of new mitochondria [5]. Studies have shown that lactate can induce mitochondrial biogenesis in rat L6 cells [6], chronic post-exercise lactate consumption, in mice, increased monocarboxylate transporter 1 (MCT1) contents in glycolytic fibers [7], and furthermore, mitochondrial adaptations caused by high-intensity interval training were attenuated after decreasing lactate accumulation in response to chronic dichloroacetate administration [8]. Mitochondrial biogenesis, the regulatory event required for the improvement of the mitochondrial network, can result in increased oxidative capacity and increased fatigue resistance [9]. Mitochondrial homeostasis is heavily regulated by peroxisome proliferator-activated receptor gamma coactivator-1α (PGC-1α), a transcriptional coactivator central to the upregulation of mitochondrial biogenesis in response to exercise [10,11]. PGC-1α expression also leads to improved mitochondrial metabolism by increasing the expression of mitochondrial enzymes in the electron transport chain, including adenosine triphosphate (ATP) synthase and Cytochrome C [12,13]. This has been purported as a possible mediator through which lactate can upregulate mitochondrial biogenesis and thereby enhance aerobic capacity, and potential exercise performance [4]. Lactate can upregulate the expression and content of proteins upregulated by PGC-1α, including MCT1 contents in glycolytic muscle [7], cytochrome c oxidase complex IV [14] and pyruvate dehydrogenase kinase (PDK) isoforms (inhibiting the conversion of lactate to pyruvate) and UCP3 (uncoupling protein 3, which improves mitochondrial oxidative capacity) [15]. PGC-1α seems to also reciprocally regulate lactate through the control of lactate dehydrogenase (LDH) isoform changes. This in part may potentiate beneficial effects of exogenous lactate in that it may be preferentially turned into pyruvate for use in aerobic metabolism, along with upregulating mitochondrial biogenesis [16,17]. This may result in improvements in exercise endurance capacity. Additionally, lactate shuttle theory also suggests that lactate can restore optimal blood pH, and enhance pyruvate yield, therefore fueling aerobic metabolism. Furthermore, increased net lactate turnover and utilization in response to exercise [18], increased MCT isoforms in skeletal muscle and cardiac tissue [19], provision of an alternative aerobic energy source via liver gluconeogenesis and the transportation and metabolism of lactate in slow-twitch oxidative muscle fibers, enhancing excitability and limiting fatigue [20], have all been reported in the literature.

Functional foods that may mimic, or even potentiate, positive adaptations of exercise, have a part to play in a holistic health approach. Oyster is considered a super food, rich in zinc, iron, calcium, selenium and numerous other vitamins, and also contains high levels of taurine [21]. Furthermore, oyster administration, prepared in many different forms, has been shown to exert antihypertensive effects on Sprague Dawley rats [22] and more recently, anti-osteoclastogenic potential [23]. The fermentation of oyster, by a highly specialized lactic acid fermentation process using *Lactobacillus brevis BJ20*, bio-converts glutamic acid within the oyster into γ-aminobutyric acid (GABA) and creates a high content of lactate in the finished product [24,25]. We previously found GABA to be associated with the release of muscle-related growth factors with increasing serum brain-derived neurotrophic factor (BDNF) levels after consuming *Lactobacillus brevis BJ20*-fermented sea tangle (*Laminaria Japonica*) [26,27]. Lactate may be considered a functional component of fermented oyster capable of explaining many of the health benefits derived from fermented foods. The bioactive capacities of functional food-derived lactate have been shown recently, including lowering innate response activation and proinflammatory cytokine levels, and inducing changes in metabolic pathways (such as reducing glycolysis rate) [28] and improving muscle differentiation and myotube hypertrophy [29]—but without elucidation on the mechanisms behind these effects.

Considering the sparse, but promising, research in the area of fermented functional foods, and particularly oyster, there has not yet been a clear mechanism proposed to account for the prophylactic effects offered through FO supplementation. Therefore, the aim of this study was to investigate the in vitro and in vivo effects of FO on skeletal muscle and endurance performance respectively, to establish whether FO may be considered an efficacious and viable supplement for healthy muscle growth and improving aerobic capacity—particularly in comparison with taurine, which has been shown to enhance skeletal muscle mitochondrial function [30] and improve human endurance performance [31].

## 2. Materials and Methods

### 2.1. Preparation of FO

**Figure 1:**
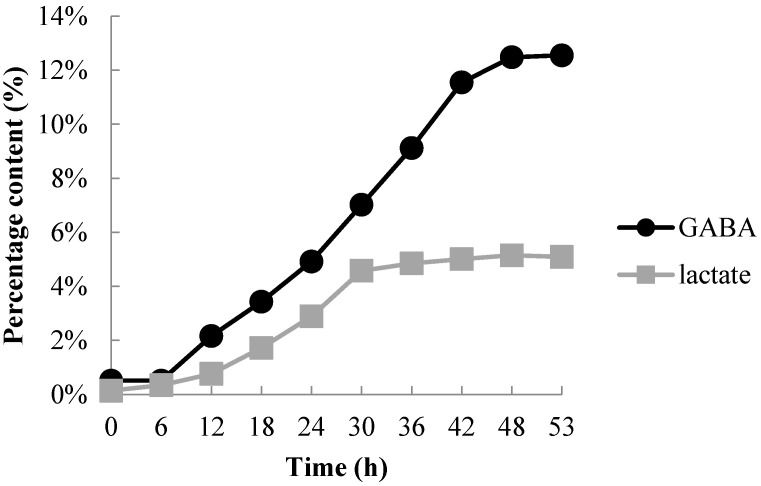
Changes of gamma amino butyric acid and lactate contents during fermentation of hydrolyzed oyster as affected by fermentation time (h).

The FO, obtained from Marine Bioprocess Co. Ltd. (Busan, Korea), was prepared using a previous procedure with slight modifications [23]. Peeled oysters, bought from Deokyeon Seafood Co. Ltd. (Tongyeong, Korea), were initially washed with tap water, homogenized, and hydrolyzed at 60 ± 50 °C for 4 h with alcalase (Alcalase^®^ 2.4 L, FG, Brenntag Korea Co., Ltd., Seoul, Korea). A vibrating sieve was used to filter the hydrolyzed oysters (120 mesh, BUCHI Labortechnik GmbH, Essen, Germany) and concentrated using a rotary evaporator (BUCHI Labortechnik GmbH). The FO manufacturing process involved *L. brevis* BJ20 (Accession No. KCTC 11377BP) being inoculated into a sterilized seed medium (yeast extract 3%, glucose 1%, monosodium glutamate 1%, water 95%) previously autoclaved at 121 °C for 15 min. After that, 10% (*v*/*v*) of the seed medium cultured at 37 °C for 24 h was inoculated into the culture medium (yeast extract 2%, glucose 2%, L-glutamic acid 2%, monosodium glutamate 3%, hydrolyzed oyster extract 50%, water 41%) and sterilized (Figure 1).

As a result of this fermentation process, a higher content of both GABA and lactate are produced (Figure 1). The mixture was filtered after fermentation at 37 °C for 48 h, and the filtrate was concentrated and then spray-dried to produce a powder sample of FO, which is composed of 46 g/100 g carbohydrate, 36 g/100 g crude protein, 6.3 g/100 g sugars and 40.3 mg/g lactic acid. Prior to use in the experiments, the FO was diluted with cell culture medium to adjust the final treatment concentrations.

#### Chemical Analysis of FO

The amounts of nutrients, such as carbohydrates, crude proteins and sugars, were determined by the methods of the Food Code of Korea (MFDS, 2019). 

**Figure 2:**
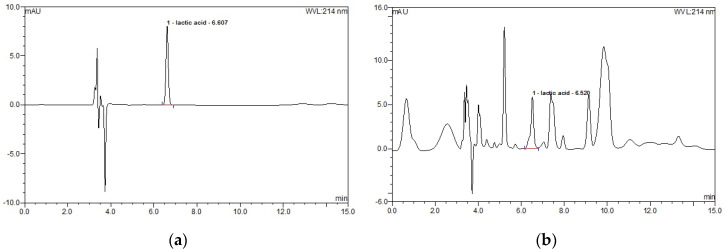
Chromatograms of standard lactic acid (**a**) and fermented oyster extract (**b**).

The content of lactic acid was determined by the citric acid analysis method of the Health Functional Food Code of Korea, 2019 [32], with slight modifications. Briefly, high-performance liquid chromatography (HPLC, Dionex U3000; Thermo Sci., Sunnyvale, CA, USA) equipped with a UV detector (Agilent TechnologiesInc., Palo Alto, CA, USA) was used to determine the content of lactic acid (Figure 2). Mobile phase is a 0.2 phosphate buffer solution (pH 2.4). Isocratic elution was conducted for 15 min at 214 nm. Injection volume was 20 μL. The solvent flow rate was 0.8 mL/min. All other chemicals used were of analytical grade.

### 2.2. In Vitro Study

#### 2.2.1. Cell Culture and Differentiation

Mouse myoblast C2C12 cells (ATCC, CRL-1772 cell line) were maintained in growth media consisting of 10% fetal bovine serum (FBS) (Corning, Woodland, CA, USA), 1% penicillin-streptomycin (PAA, Austria) and Dulbecco’s Modified Eagle’s Medium (DMEM) (Corning, USA) in a 37 °C, 5% CO_2_, humidified atmosphere. Cell differentiation was carried out once the cells were grown to 100% confluence and then induced to differentiate after switching them to a differentiation medium consisting of DMEM containing 1% horse serum (H0146, Sigma-Aldrich, St. Louis, MO, USA) every 24 h, and harvested at a 96 h time point. Cells were incubated in DMEM supplemented with 10% FBS and 1% penicillin-streptomycin. The experimental design has been outline in Table 1.

#### 2.2.2. Enzyme-Linked Immunosorbent Assay (ELISA) Analysis

Lactate content, Glycogen content and ATP activity in C2C12 muscle cells treated with FO were examined with ELISA kits (ECLC011, BioAssay Systems, Hayward, CA, USA). The glycogen concentration was measured using BioAssay Systems, E2GN005 (BioAssay Systems, Hayward, CA, USA), and ATP concentration using (ATP Assay Kit #K354-100, BioVision, Milpitas, CA, USA).

#### 2.2.3. Western Blot Analysis

For Western blot analysis, cells were homogenized in a cell lysis buffer (200 μL) with 30 min interval ice incubation, and then centrifuged at 14,000 rpm, at 4 °C for 20 min. Protein concentrations were determined using the Bradford Protein Assay Kit. The proteins were then resolved by SDS-PAGE (10%) (Sodium dodecyl sulfate-Polyacrylamide Gel Electrophoresis), transferred to a nitro cellulose membrane using the semi-dry transfer system (Biorad, Hercules, CA, USA) and treated with a blocking solution (0.5% skim milk, 1 × Phosphate-buffered saline with Tween 20 (PBST) buffer) for 1 h. The membrane was then washed three times for 10 min in PBST buffer, incubated overnight (4 °C) with primary antibodies against total-AMPK, p-AMPK (Thr172) (Santa Cruz Biotechnology, Dallas, TX, USA) LDH (Santa Cruz, CA, USA), SDHA (Santa Cruz, CA, USA) and PGC-1α (Santa Cruz, CA, USA), and then washed again with the aforementioned procedure. Following this, the membranes were probed with secondary antibodies and washed with PBST buffer. After treatment with the buffer, protein band intensity was detected using the enhanced chemiluminescence Chemi-Doc, XRS system (BIORAD, Hercules, CA, USA) and compared with Glyceraldehyde 3-phosphate dehydrogenase (GAPDH) and total-AMPK after activation using a Western blot detection kit (Biorad, USA).

#### 2.2.4. Measurement of Cell Viability

**Figure 3:**
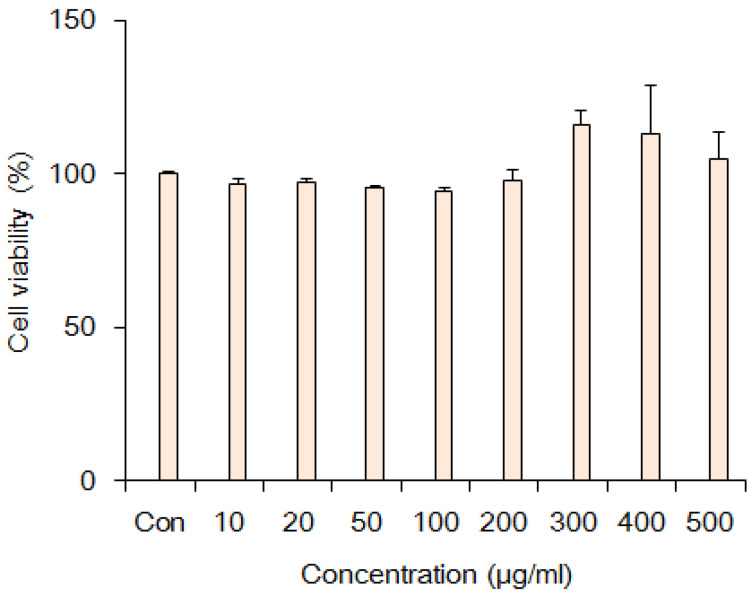
Cytotoxicity of Fermented oyster extracts on cell viability of C2C12 myoblast. Data were shown as mean ± SD (standard deviation) for groups of three experiments.

To determine the cytotoxicity of FO on C2C12 muscle cells, we utilized the Water-soluble tetrazolium-1 (WST-1) method. Cells were seeded at 0.5 × 10^4^ cells in 96-well plates and preincubated for 24 h. C2C12 muscle cells were treated with different concentrations of FO (10, 50 μg/mL) for 24 h. After the treatment period, WST-1 was added to each well and incubated for 1 h at 37 °C. The absorbance was measured at 460 nm using a microplate reader (Molecular Devices, San Jose, CA, USA) (Figure 3).

#### 2.2.5. Myogenic Differentiation 1 (MyoD) mRNA Expression

The expression level of MyoD is changed according to the induction time of C2C12 muscle cells [33]. In order to identify the time point that is most expressed in this experiment, induction of differentiation was conducted for 48, 96 and 168 h. As a result, the expression of MyoD mRNA was most significantly increased at 96 h. Based on this experiment, the differentiation cell was secured at 96 h after induction of differentiation and the experiment was conducted.

### 2.3. In Vivo Exercise Study

#### 2.3.1. Animals

Five-week-old male C57BL/6J mice weighing 18–23 g were purchased from Samtako BIOKOREA (Gyeonggi-do, Osan, Korea) and housed in a regulated environment (temperature, 22 ± 3 °C; relative humidity, 50% ± 10%; 12 h light/dark cycle beginning at 07:00) [27]. All experiments were conducted in accordance with the guidelines of the Southeast Medi-Chem Institute (SEMI, Institutional Animal Care and Use committee) (ethical approval number: SEMI-16–02). As described in Table 2, 10 mice were assigned to each group. Excluding the CON (control) group, all other groups were exercised via the Rota-rod method.

#### 2.3.2. Rota-Rod Test

The Rota-rod test method used in the present study was adapted from a previous study by Rho et al. [34]. In this experiment, the Rota-rod consisted of five cubicles with a diameter of 7 cm, a spacing of 15 cm and a rotatable gyratory rod with a height of 60 cm (LSi Letica Rota-Rod R/S). The result of this test is the mean time the animal is able to remain on top of the gyratory rod—each animal performed three trials to ascertain mean values. The Rota-rod gradually increased in rpm as the test progressed, starting from 0 rpm and continuing to increase until the mice fall off the gyratory rod. Mice were pre-trained one week before testing, at a speed of 5 rpm.

#### 2.3.3. Western Blot Analysis

The proteins were then resolved by SDS-PAGE (10%) (Polyacrylamide Gel Electrophoresis), transferred to a polyvinylidene fluoride membrane using the semi-dry transfer system (Bio-Rad, USA) and treated with a blocking solution (5% skim milk, 1 × TBST buffer) for 1 h. The membrane was then washed three times for 10 min in TBST, incubated overnight (4 °C) with primary antibodies against MYH2, p-AMPK (Thr172), total-AMPK, PGC-1α, cytochrome C and UCP3 and then washed again with the aforementioned procedure. After treatment with the buffer, protein band intensity was detected using enhanced chemiluminescence (Chemi-Doc, BIORAD XRS system) and compared with GAPDH and total-AMPK after activation using a Western blot detection kit (West Save Gold, Abfrontier, Seoul, Korea).

#### 2.3.4. ELISA Analysis

Serum: The collected blood was coagulated at room temperature for 30 min and centrifuged at 3000 rpm at 4 °C for 15 min. The separated blood supernatants were measured for glucose using an ELISA kit (BioAssay Systems, USA).

Muscle and liver tissue: The absorbed muscle tissue and liver tissues were perfused with the respective buffers corresponding to the measurement factors and the absorbance was measured. Citrate, Glycogen, L-Lactate, Lactate dehydrogenase (LDH) (BioAssay Systems, USA), ATP and succinate dehydrogenase (SDH) activity (Bio Vision, San Francisco, CA, USA) was identified.

#### 2.3.5. Statistical Analysis

Data presented are mean ± standard deviation (SD). All analyses were performed using Statview software. The effect of treatment on performance, biochemical and immunohistochemical data were analyzed by one-way analysis of variance (ANOVA) followed by post hoc analysis using the Fisher’s post-hoc least significant differences (PLSD) test.

## 3. Results

### 3.1. In Vitro Study

**Figure 4:**
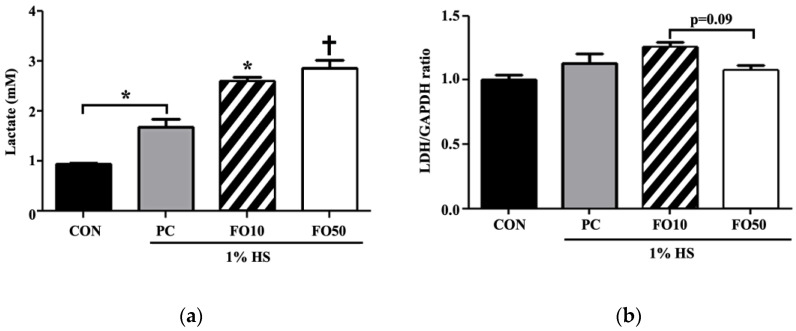
Changes in (**a**) lactate and (**b**) lactate dehydrogenase levels were determined in C2C12 muscle cells following administration of 10 and 50 μg/mL of FO. Each bar represents the mean ± standard deviation (SD). A one-way analysis of variance (ANOVA) test was carried out to determine significant differences (* *p* < 0.05, ^†^ *p* < 0.001) between groups.

**Figure 5:**
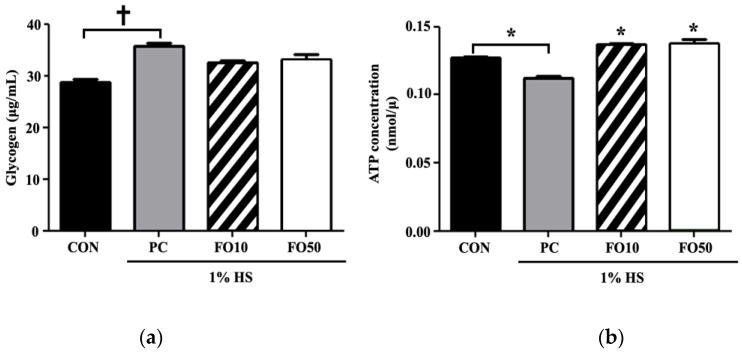
Changes in (**a**) glycogen and (**b**) Adenosine triphosphate (ATP) levels were determined in C2C12 muscle cells following administration of 10 and 50 μg/mL of FO. Each bar represents the mean ± SD. A one-way ANOVA test was used to determine significant differences (* *p* < 0.05 vs. positive control, † *p* < 0.001 vs. positive control).

**Figure 6:**
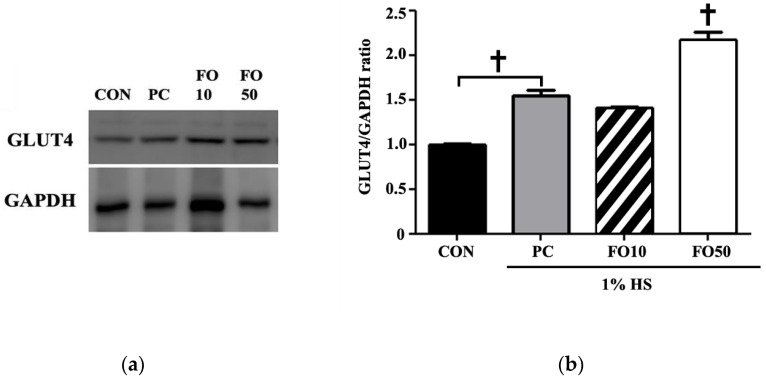
(**a**) image of Western blot analysis. (**b**) Changes in GLUT4 levels were determined in C2C12 muscle cells following administration of 10 and 50 μg/mL of FO. Each bar represents the mean ± SD. A one-way ANOVA test was carried out to determine significant differences (^†^ *p* < 0.001 vs. positive control).

**Figure 7:**
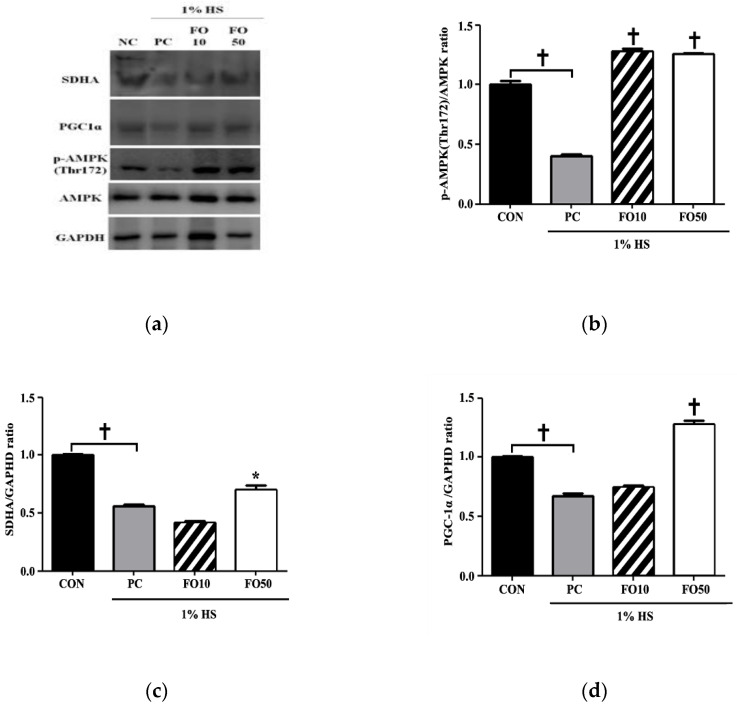
(**a**) image of Western blot analysis. Changes in (**b**) phospho-adenosine monophosphate-activated protein kinase (p-AMPK), (**c**) succinate dehydrogenase (SDHA) and (**d**) peroxisome proliferator-activated receptor gamma coactivator-1 α (PGC-1α levels were determined in C2C12 muscle cells following administration of 10 and 50 μg/mL of FO. Each bar represents the mean ± SD. A one-way ANOVA test was carried out to determine significant differences (* *p* < 0.05 vs. positive control, ^†^ *p* < 0.001 vs. positive control).

Biochemical and Western blot analysis: Biochemical analysis of myoblast C2C12 cells administered with FO10 and FO50 demonstrated a significant increase in lactate concentration in a dose-response manner, *p* < 0.05 and *p* < 0.001, respectively (Figure 4). LDH concentration tended toward an increase in the FO10 group, but was unchanged in the FO50 group, compared to CON and PC. Thus, FO demonstrated the ability to increase the concentration of a significant bioactive molecule, lactate. Glycogen and ATP concentrations were also measured, with the latter being significantly increased by both FO10 and FO50 (*p* < 0.05) (Figure 5). Glycogen was largely unchanged in both experimental groups. Furthermore, concerning energy metabolism, the glucose transporter protein, GLUT4, was significantly increased as a result of FO50 administration (*p* < 0.001) (Figure 6). Western blot analysis measured important regulators of mitochondrial homeostasis and found that PGC-1α was significantly upregulated by FO50 administration (*p* < 0.001). FO10 and FO50 were both capable of significantly increasing the concentration of p-AMPK (Thr172), *p* < 0.001 (Figure 7).

### 3.2. In Vivo Exercise Study

#### 3.2.1. Rota-Rod Test

**Figure 8:**
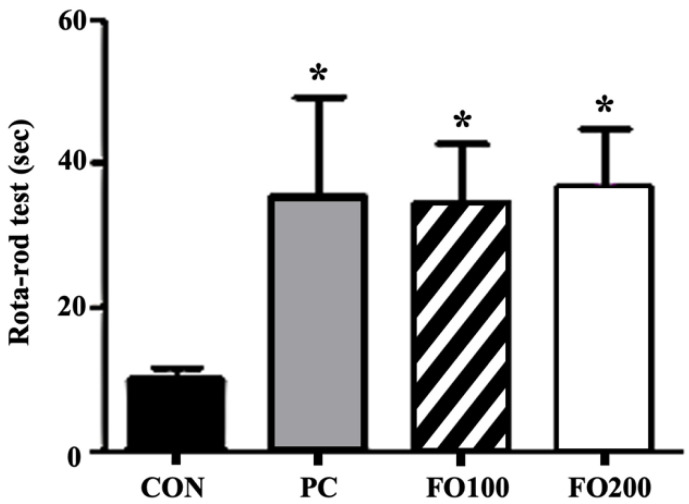
Exercise endurance was measured as the total time sustained on the gyratory rod. Differences in total time were recorded following administration of 100 and 200 μg/mL of FO. Each bar represents the mean ± SD. A one-way ANOVA test was carried out to determine significant differences (* *p* < 0.05 vs. Control).

The Rota rod test was utilized to assess the physical performance of mice following the supplementation of FO. Both the PC group (35.7 ± 39 s, *d* = 0.9—indicating a large effect size), FO100 (34.8 ± 25 s, *d* = 1.4) and FO200 (36.8 ± 25.8 s, *d* = 1.4) experimental groups demonstrated a significantly improved exercise performance compared to the control group (10.1 ± 4.4 s) (*p* < 0.05) (Figure 8). FO seems to provide performance-enhancing effects comparable to those provided by supplements such as Taurine (PC group).

#### 3.2.2. Biochemical Analysis

**Figure 9:**
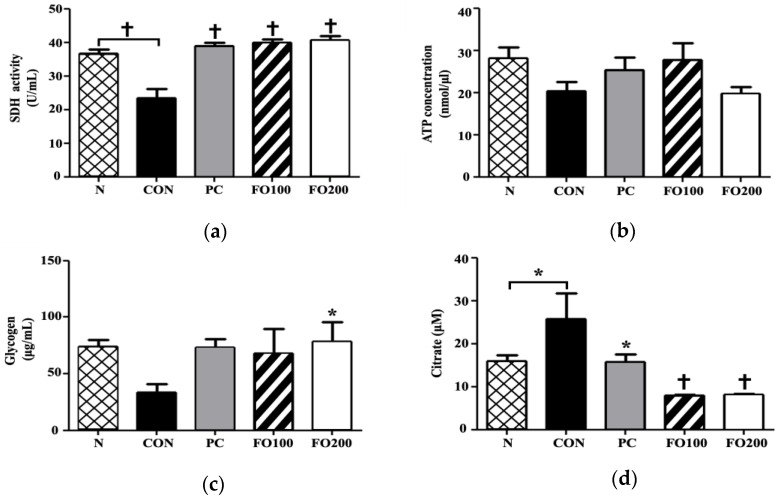
Changes in muscle (**a**) SDH, (**b**) ATP, (**c**) glycogen and (**d**) citrate were measured following administration of 100 and 200 μg/mL of FO. Each bar represents the mean ± SD. A one-way ANOVA test was carried out to determine significant differences (* *p* < 0.05 vs. Control, ^†^ *p* < 0.001 vs. Control).

Muscle: Concerning energy storage and metabolism, the present study showed that FO100 and FO200 ingestion helped to restore levels of SDH to pre-test values. ATP concentration did not show a significant change following supplementation; however, glycogen stores were increased as a result of FO200 supplementation (*p* < 0.05) (Figure 9). Interestingly, citrate levels were significantly reduced as a result of FO100 and FO200 ingestion (*p* < 0.001), which could indicate energy production favoring glycolysis.

**Figure 10:**
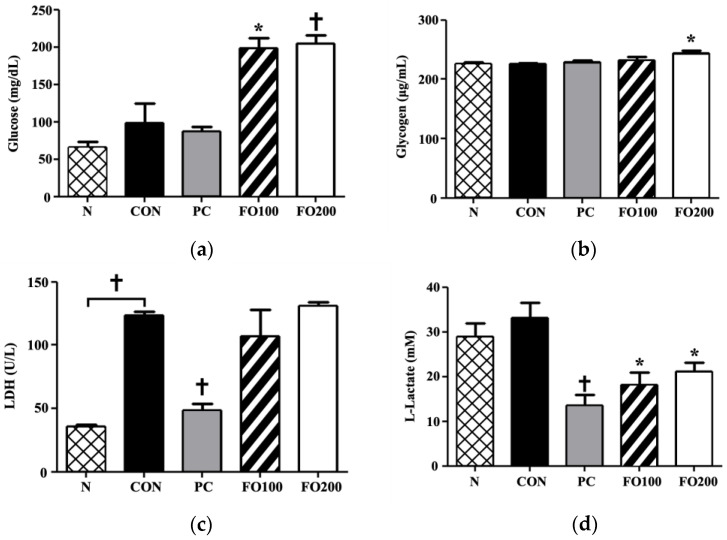
Changes in serum (**a**) glucose and liver (**b**) glycogen, (**c**) LDH and (**d**) L-lactate were measured following administration of 100 and 200 μg/mL of FO. Each bar represents the mean ± SD. A one-way ANOVA test was carried out to determine significant differences (* *p* < 0.05 vs. Control, ^†^ *p* < 0.001 vs. Control).

Serum and liver: To further analyze the effects of FO on energy production and storage, we measured the concentration of glucose in the blood serum and glycogen in the liver. Glucose levels were significantly increased as a result of FO100 and FO200 supplementation (*p* < 0.05 and *p* < 0.001, respectively) (Figure 10). FO200 was able to significantly increase the levels of glycogen in the liver (*p* < 0.05), while FO100 tended towards an increase. All of these improvements seemed to occur on a dose-response basis. FO100 and FO200 prevented levels of LDH from being depleted, as was seen in the PC group. L-lactate was significantly reduced in the FO100 and FO200 groups (*p* < 0.05).

**Figure 11:**
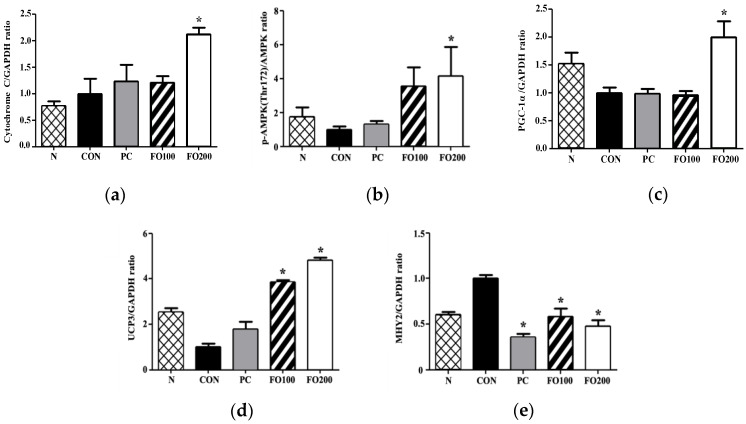
Immunohistochemical analysis of (**a**) cytochrome C, (**b**) p-AMPK (Thr172), (**c**) PGC-1a, (**d**) UCP3 and (**e**) MYH2 was carried out to measure changes following administration of 100 and 200 μg/mL of FO. Each bar represents the mean ± SD. A one-way ANOVA test was carried out to determine significant differences (* *p* < 0.05 vs. Control).

Immunohistochemistry: Immunohistochemical analysis in vivo shows that administration of FO200 led to a significant increase in cytochrome C levels (*p* < 0.05). There were also significantly higher levels of p-AMPK in the FO200 group (*p* < 0.05), with a tendency to increase in the FO100 group. A significant increase in PGC-1α levels also occurred in the FO200 group (*p* < 0.05) (Figure 11). The increase in these mitochondrial homeostasis markers, particularly pAMPK and PGC-1α, supports the results found in vitro. UCP3 levels were also significantly increased in both the FO100 and FO200 groups, in a dose-dependent fashion (*p* < 0.05).

## 4. Discussions

Fermented oyster is considered a super food rich in vitamins and minerals, and containing high levels of taurine [21]. Supplementation of fermented oyster has been reported to provide a variety of prophylactic benefits, including antihypertensive effects [22] and more recently, anti-osteoclastogenic potential [23]. A specialized fermentation process, using *Lactobacillus brevis* BJ20, enriches the lactate, as well as gamma aminobutyric acid (GABA), content and enhances the bioactive properties of oyster. We sought to be the first to determine whether FO administration in vitro and supplementation in vivo can improve skeletal muscle adaptive responses to endurance training and endurance performance, respectively. The results of the present study demonstrate that, in vitro, mitochondrial homeostasis regulator PGC-1α was significantly upregulated by FO50 administration alongside the upregulation of upstream p-AMPK required for the phosphorylation of PGC-1α (*p* < 0.001). Lactate concentration was also significantly increased in C2C12 cells (*p* < 0.001). Much like our in vitro results, in vivo supplementation of FO200 significantly increased cytochrome C, p-AMPK and also PGC-1α content in muscle (*p* < 0.05). In terms of muscle energy production, citrate levels were suppressed (*p* < 0.05), SDH activity was increased (*p* < 0.001) and glycogen storage was also increased following FO supplementation (*p* < 0.05). These results have identified FO as an exogenous stimulant of SkM mitochondrial biogenesis, and a functional food ingredient containing bioactive properties, such as lactate and GABA, able to potentiate the adaptive responses to exercise, while also offering a glimpse into potential mechanisms by which these effects may occur.

Endurance training alone induces beneficial physiological adaptations, including remodeling of the mitochondrial network, contractile apparatus and vasculatures [1,2], while helping to maintain muscle mass, regeneration and hypertrophy [35]. A mouse muscle precursor cell line, namely C2C12 cells, may be induced to differentiate into contracting myotubes expressing muscle-specific proteins [29], and therefore lends itself well as a model for muscle satellite cells. Mitochondria, being the biological engine of the cell, improves in quantity and quality in response to endurance training, and this mitochondrial remodeling is central to the improvement of skeletal muscle contractile and metabolic functions. Upregulation of PGC-1α in response to FO administration, in vitro (Figure 7), implies an upregulation of mitochondrial biogenesis [10]. The concomitant rise in p-AMPK expression (Figure 7) may indicate the signaling pathway involved. Kitaoka et al. reported increased PGC-1α mRNA expression in mouse skeletal muscle following lactate administration, whereas Hashimoto et al. hypothesized a mediator role for oxidative stress in the lactate-induced adaptations observed in L6 cells [14,15]. The potential metabolic stress environment induced by endogenous lactate may also be related to stress kinase p38MAPK regulation of mitochondrial biogenesis by regulating expression of PGC-1α in muscles [36], and AMPK can activate p38MAPK [37]. Although our study did not investigate this intermediary signaling pathway step, we can deduce a progression towards mitochondrial biogenesis following FO administration. An interesting corroboration of these results in vivo show FO200 supplementation, accompanied with exercise, to significantly increase PGC-1α and p-AMPK expression in muscle; however, neither exercise alone nor the addition of taurine supplementation had the same effects (Figure 11). This is interesting as Ommati et al. put forward that the positive effects on performance and muscle function seen in taurine were most likely related to taurine’s ability to modulate cellular mitochondrial function [30]. Mechanistically, taurine seems to regulate mitochondrial ability through sequestering Ca^2+^, whereas FO may act through the upregulation of PGC-1α. It has been well established that exercise-induced PGC-1α expression in muscle leads to changes toward oxidative muscle fiber-type, increased angiogenesis, robust mitochondrial biogenesis and oxidative phosphorylation and even fatigue resistance [37,38,39]. However, the present study’s use of Rota-rod exercise alone may not have been a high enough intensity, or duration, to induce significant changes in cytochrome C, PGC-1α and p-AMPK expression in muscle, even though they were significantly increased in FO200—as it has been shown that training volume is a key factor affecting training-induced mitochondrial biogenesis [40,41]. This may imply that supplementation of FO, regardless of low exercise intensity, can produce desirable physiological responses pertaining to mitochondrial biogenesis—this being particularly beneficial to the aging population. Furthermore, we also demonstrated that taurine administration improved Rota-rod test performance tantamount to the improvements seen in FO experimental groups. Such results indicate that FO supplementation may be considered an ergogenic aid utilized in the same manner as taurine—for performance improvements [31]. Another interesting finding was the significant increase in cytochrome C levels following FO200 supplementation. Located in the mitochondrial inner membrane, cytochrome C functions as an electron shuttle in the electron transport chain, vital in the production of ATP. In order for the mitochondria to satisfy the energy requirements of the cell during exercise, each cytochrome C molecule must continue to shuttle electrons from respiratory complex III to complex VI of the electron transport chain. Wu et al. reported that overexpression of PGC-1α in myoblasts was able to increase cytochrome C protein levels as an adaptation to facilitate increased oxygen utilization [10]. Thus, FO is shown here to increase mitochondrial energy production and efficiency following exercise, in a dose-dependent manner.

Concerning glucose breakdown and mitochondrial energy metabolism, FO10 and FO50 were both able to significantly increase ATP concentration in C2C12 cells (Figure 5). The aforementioned rise in p-AMPK may have a role to play in increasing glycolytic rate, as AMPK has been shown to induce glycolysis by directly phosphorylating 6-phosphofructo-2-kinase (PFK2) in muscles, which by activating PFK1, enhances glycolytic rate [42]. This may ultimately lead to metabolic adaptation and cell survival, particularly in human cells exposed to oxidative stress. Although changes in ATP levels were statistically insignificant, FO100 did cause there to be greater ATP production. In addition, citrate was significantly reduced, and succinate dehydrogenase levels were increased by both FO100 and FO200 (Figure 9). High levels of cytosolic citrate, produced in the Krebs cycle from oxaloacetate, are known to inhibit the rate-limiting glycolytic enzymes, PFK1 and PFK2 [43]. Mitochondrial citrate can also inhibit pyruvate dehydrogenase and SDH [44]. Taken together, a reduction in citrate levels favors glycolysis, coinciding with the current findings of greater ATP production in vitro and hence an increase in glycolytic capacity. Furthermore, the significant rise in the glucose transporter protein, GLUT4, may indicate a greater rate of glucose uptake to be utilized in glycolytic energy production (Figure 6). A potential mechanism may involve hypoxia-inducible factor (HIF-1 transcription factor), which increases during high-intensity exercise and is closely associated with lactate transport production and metabolism [44].

In terms of FO’s effects on myogenesis, our study reported reduced protein expression of MYH2 following FO administration in vivo. An explanatory model via a lactate mechanism may be considered regarding these results. The decrease in MYH2 expression, a marker of late differentiation phase myogenesis, was previously reported by Willkomm et al. in C2C12 cells in response to lactate administration [45]. However, this was coupled with increased early differentiation phase marker expression and apparent cell-cycle withdrawal. Furthermore, Tsukamoto et al. reported that lactate increases the transcription of specific myosin heavy-chain isoforms, demonstrating that MYH2 expression levels were unchanged by lactate [29]. Another implication of this result stems from MYH2 being a marker of myogenesis typically in response to muscle damage and may indicate that FO acts to protect against exercise-induced skeletal muscle damage. Further investigation into the effects of FO on early phase differentiation may reveal whether late-stage withdrawal occurs and help to ascertain the likelihood of lactate being a causal factor.

Liver glucose increases as a result of exercise via two main processes, glycogenolysis and gluconeogenesis. During prolonged exercise, gluconeogenesis is upregulated to compensate for the decline in liver glycogen stores and help to maintain euglycemia. As demonstrated by our results, there was a marginal increase in hepatic glucose following exercise in the CON group, however FO100 and FO200 were both able to further, and significantly, increase glucose production in the liver, with the latter significantly increasing glycogen stores (Figure 10). It is well known that exercise induces a state of hypoglycemia, and chronic exercise helps the body become more resistant by improving hepatic gluconeogenic capacity [46,47]. Our results indicate that FO may be supplemented to increase hepatic energy production, and therefore aid the body in replenishing energy stores and offsetting hypoglycemia. The data from the present study does not allow us to make any clear mechanistic conclusions, however it is reasonable to suggest that a rise in circulating lactate would provide the liver with more gluconeogenic substrate [48], or that a lactate-induced increase in PGC-1α expression, in vivo and in vitro, can promote hepatic gluconeogenic gene expression [49].

## 5. Conclusions

Fermented foods have been demonstrated to provide a host of prophylactic effects on health and longevity. Fermented oyster is relatively new in this field and presents as a health-promoting functional food while also serving as a source of exogenous lactate. The present study demonstrates the efficacy of FO as a supplementation for health, and even an ergogenic aid potentiating exercise performance benefits and providing upregulation of vital components of mitochondrial metabolism in skeletal muscle. A lot more investigation is required to elucidate the mechanisms by which FO, lactate and other constituents (including GABA) of FO may act to provide enhancing effects of health and exercise performance, namely, dose-response, potential myogenic effects, interaction with HIF-1 and a progression to human studies.

Lactate, particularly, seems to be an explanatory source of the health benefits of FO. Furthermore, such a claim may be put forward in light of previous studies demonstrating that lactate infusion at rest can increase plasma BDNF concentrations in humans [50], and that a mechanism may exist for the involvement of lactate in the promotion of C2C12 cell differentiation and myotube hypertrophy [29]. FO supplementation can seemingly upregulate key components of mitochondrial biogenesis and markers of myogenesis in vitro. These findings are fairly consistent in vivo and demonstrate a potentiating effect of FO on the adaptive responses of exercise on skeletal muscle and energy metabolism—with lactate being a key bioactive molecule.

The present study does have limitations mainly due to it being a preliminary investigation into FO, lactate and exercise performance. More robust methods of statistical analyses could have been used to control for multiple analysis, such as Bonferroni or Scheffé. To improve test repeatability and reliability, we will utilize these methods in subsequent FO studies. Furthermore, the Rota-rod test, used to evaluate exercise endurance performance, may not only have been too low of an intensity to impact mitochondrial biogenesis, but too brief of a test to claim any effects on aerobic performance. Nonetheless, our results do indicate a performance improvement following FO administration coupled with improvements in endurance performance indicators, thus warranting further investigation into the extent, and nature, of performance improvements possible following FO intake. The specificity of our tests must be reviewed in future studies.

## Figures and Tables

**Table 1:** In vitro experimental groups.

Group	Differentiation	Treatment	Harvest Time
CON	1% Horse Serum	No treatment	
PC		1% Horse Serum	96 h
FO10		1% Horse Serum + Fermented Oyster (10 μg/mL)	After co-treatment 96 h
FO50		1% Horse Serum + Fermented Oyster (50 μg/mL)	

CON (control, no treatment), PC (positive control, 1% horse serum), FO10 (fermented oyster, 10 μg/mL) and FO50 (fermented oyster, 50 μg/mL).

**Table 2:** In vivo experimental groups.

Group	Dose (mg/kg)	Treatment		Number
**CON**		Saline		10
**Ex**			Exercise	10
**PC**	500	Taurine		10
**FO100**	100	Fermented Oyster		10
**FO200**	200			10

CON (control), Ex (exercise), PC (positive control, exercise + Taurine 500 mg/kg per os (orally)), FO100 (Exercise + FO100 mg/kg per os (orally)), and FO200 (Exercise + FO200 mg/kg per os (orally)).
